# 5-Hydroxymethylcytosine Profiles Are Prognostic of Outcome in Neuroblastoma and Reveal Transcriptional Networks That Correlate With Tumor Phenotype

**DOI:** 10.1200/PO.18.00402

**Published:** 2019-05-16

**Authors:** Mark A. Applebaum, Erin K. Barr, Jason Karpus, Ji Nie, Zhou Zhang, Amy E. Armstrong, Sakshi Uppal, Madina Sukhanova, Wei Zhang, Alexandre Chlenski, Helen R. Salwen, Emma Wilkinson, Marija Dobratic, Robert Grossman, Lucy A. Godley, Barbara E. Stranger, Chuan He, Susan L. Cohn

**Affiliations:** ^1^ University of Chicago, Chicago, IL; ^2^ Texas Tech University Health Sciences, Lubbock, TX; ^3^ Northwestern University, Chicago, IL; ^4^ Indiana University, Indianapolis, IN; ^5^ Howard Hughes Medical Institute, Chevy Chase, MD

## Abstract

**PURPOSE:**

Whole-genome profiles of the epigenetic modification 5-hydroxymethylcytosine (5-hmC) are robust diagnostic biomarkers in adult patients with cancer. We investigated if 5-hmC profiles would serve as novel prognostic markers in neuroblastoma, a clinically heterogeneous pediatric cancer. Because this DNA modification facilitates active gene expression, we hypothesized that 5-hmC profiles would identify transcriptomic networks driving the clinical behavior of neuroblastoma.

**PATIENTS AND METHODS:**

Nano-hmC-Seal sequencing was performed on DNA from Discovery (n = 51), Validation (n = 38), and Children’s Oncology Group (n = 20) cohorts of neuroblastoma tumors. RNA was isolated from 48 tumors for RNA sequencing. Genes with differential 5-hmC or expression between clusters were identified using DESeq2. A 5-hmC model predicting outcome in high-risk patients was established using linear discriminant analysis.

**RESULTS:**

Comparison of low- versus high-risk tumors in the Discovery cohort revealed 577 genes with differential 5-hmC. Hierarchical clustering of tumors from the Discovery and Validation cohorts using these genes identified two main clusters highly associated with established prognostic markers, clinical risk group, and outcome. Genes with increased 5-hmC and expression in the favorable cluster were enriched for pathways of neuronal differentiation and KRAS activation, whereas genes involved in inflammation and the PRC2 complex were identified in the unfavorable cluster. The linear discriminant analysis model trained on high-risk Discovery cohort tumors was prognostic of outcome when applied to high-risk tumors from the Validation and Children’s Oncology Group cohorts (hazard ratio, 3.8).

**CONCLUSION:**

5-hmC profiles may be optimal DNA-based biomarkers in neuroblastoma. Analysis of transcriptional networks regulated by these epigenomic modifications may lead to a deeper understanding of drivers of neuroblastoma phenotype.

## INTRODUCTION

Although the epigenetic modification 5-methylcytosine creates a repressed chromatin environment and decreased gene expression, elevated 5-hydroxymethylcytosine (5-hmC) deposition across the gene body facilitates active transcription.^1^ Cytosines containing the 5-hmC modification help establish open chromatin regions,^2^ and a spectrum of biologic processes are modulated by this epigenomic modification.^3^ A highly sensitive and robust 5-hmC sequencing approach (Nano-hmC-Seal) has been developed that allows genome-wide profiling of 5-hmC using a limited amount of genomic DNA. This technology has shown dynamic alterations in 5-hmC patterns in rare hematopoietic cell populations.^3,4^ Furthermore, Nano-hmC-Seal requires as little as 100 ng of DNA from frozen tissue, enabling analysis of clinical samples with limited available tissue. More recently, 5-hmC signatures in cell-free DNA have been generated and shown to be robust diagnostic biomarkers for adult human cancer.^5,6^

**Context**

**Key Objective**
Recently, 5-hydroxymethylcytosine (5-hmC) profiles have been developed as a diagnostic biomarker for adult malignancies. To investigate the prognostic value of this DNA modification in neuroblastoma, we applied Nano-hmC-Seal, a revolutionary, low-cost, genome-wide technology that requires minimal input DNA, to profile 5-hmC in 109 tumors.
**Knowledge Generated**
Grouping of all tumors using genes with differential 5-hmC in the Discovery cohort identified two clusters highly associated with established prognostic markers, clinical risk-group, and outcome. 5-hmC facilitates gene expression, and genes with differential 5-hmC and expression were enriched for pathways of neurodevelopment in the favorable cluster. Genes in the clinically aggressive cluster were enriched for pathways of inflammation and the PRC2 complex. Using 5-hmC levels in high-risk patients from the Discovery cohort, we developed an outcome prediction model that successfully identified events an independent cohort (n = 24) of high-risk patients.
**Relevance**
5-hmC profiling could provide information on factors that determine the clinical behavior of neuroblastoma (ie, *MYCN* status, copy number alterations, and transcriptional networks) using one simple assay. Ongoing efforts include prospectively testing the prognostic value of 5-hmC profiles from tumors and cell-free DNA.

Neuroblastoma is characterized by a broad spectrum of clinical behavior, reflecting its biologic heterogeneity.^7^ A combination of clinical and biologic prognostic markers, including age, stage, *MYCN* status, ploidy, and histology, is used to classify risk and stratify treatment of patients with neuroblastoma. Although current therapeutic strategies lead to excellent survival for children with low-risk (LR) or intermediate-risk (IR) disease, outcomes remain poor for high-risk (HR) patients, with fewer than half achieving long-term survival.^7^ Significant heterogeneity exists within HR tumors,^7^ and new biomarkers are needed to distinguish patients who will respond to standard treatments from those who may benefit from alternative approaches. Although little is known about the role of 5-hmC in the pathogenesis of pediatric cancer, we have shown increases in 5-hmC are an important component of the hypoxia response in neuroblastoma cell lines.^8^ On the basis of previous adult cancer studies, we hypothesized 5-hmC profiles would serve as robust biomarkers for children with neuroblastoma. We also anticipated transcriptional networks critical for promoting distinct neuroblastoma phenotypes would be regulated by the genomic pattern of this DNA modification.

## PATIENTS AND METHODS

### Patients and Isolation of Tumor DNA and RNA

Snap-frozen tumors were collected previously at the time of diagnosis per local tissue banking protocols at the University of Chicago (Discovery cohort) and Lurie Children’s Hospital of Chicago (Validation cohort). DNA was extracted from tissue using the Gentra Puregene Tissue kit (Qiagen, Valencia, CA) according to the manufacturer’s directions. If remaining tissue was available, RNA was extracted using TRIzol Reagent (Thermo Fisher Scientific, Waltham, MA) according to the manufacturer’s directions. Patients’ neuroblastomas were diagnosed between 1985 and 2016 and received different treatments based on era of diagnosis and risk stratification. Additional DNA samples from HR stage 4 patients were obtained from the Children’s Oncology Group (COG) neuroblastoma biobank (COG cohort) for predictive model development. Clinical data for the COG cohort were extracted from the International Neuroblastoma Risk Group database.^7^ Differences in clinical and tumor characteristics were evaluated with Fisher’s exact tests and *t* tests for categorical and continuous variables, respectively. All protocols were approved by local institutional review boards.

### Nano-5-hmC-Seal Library Preparation and Sequencing

Nano-hmC-Seal libraries were constructed from 100 ng of genomic DNA, as described.^3,4^ Fifty base-pair, paired-end libraries were sequenced on an Illumina NextSeq 500 (San Diego, CA). FASTQC, version 0.11.5, was used to assess sequence quality.^9^ Raw reads were processed with Trimmomatic^10^ and aligned to hg19 with Bowtie2, version 2.3.0 (https://sourceforge.net/projects/bowtie-bio/files/bowtie2/2.3.0/), using default settings.

### 5-hmC Distribution by Genomic Feature

Annotation of read alignments to genomic features was performed with hypergeometric optimization of motif enrichment (HOMER)^11^ using default settings with the -histone flag. HOMER normalizes raw read counts into 10 million tags per sample, allowing direct comparison between samples of multiple batches. Differences between log-transformed quantity of 5-hmC between risk groups according to genomic feature were assessed by paired *t* tests with Benjamini-Hochberg correction.^12^ CpG islands were downloaded from the University of California, Santa Cruz, genome browser, and CpG shores were defined as 2-kb regions adjacent to each CpG island. Enhancer regions were annotated from prior studies in neuroblastoma cell lines from the Enhancer Atlas^13^ and van Groningen et al.^14^

### Chromosomal Copy Number Evaluation

DNA from HR tumors was analyzed using the OncoScan Affymetrix microarray platform, following the manufacturer’s instructions (Affymetrix, Santa Clara, CA). Data were analyzed using the Chromosome Analysis Suite, version 3.3. Copy number gains and losses were defined as a relative increase or decrease from the modal number for each tumor.^15^

### RNA-Sequencing Library Preparation and Sequencing

After RNA extraction, DNA was removed with the TURBO DNA-free kit (Thermo Fisher Scientific) per the manufacturer’s instructions. Ribosomal RNA was removed with the oligo-DT kit, and a directional RNA library was constructed. Fifty base-pair, single-end libraries were sequenced on an Illumina HiSeq 4000. Reads underwent quality control, trimmed as indicated, and aligned to hg19 using STAR RNAseq aligner.^16^

### Identification of Genes With Differential 5-hmC and Expression

Aligned reads with a mapping quality score of 10 or higher were counted using featureCounts^17^ of Subread, and the -gene flag and the gencode.v27lift37.annotation.gtf file from GENCODE. Read counts of 5-hmC for the entire gene body and RNA sequencing (RNA-seq) across exons were loaded into the DESeq2 v1.20.0^18^ package in R, version 3.5.0 (R Project, https://cran.r-project.org/bin/windows/base/old/3.5.0/) with a model that adjusted for sex and batch. *P* < .05 after multiple testing adjustment was considered significant,^12^ and genes were filtered for those with log-two–fold change in the top 10% of each cluster. Pheatmap, version 1.0.10 (R Project), was used to determine the distance matrix between samples for hierarchical clustering. Pathway analysis was performed with XGR, version 1.1.3,^19^ for datasets in MSigDB.^20^ Genes used for pathway analysis only included those with increased 5-hmC and expression within each respective cluster.

### Generation and Validation of an Outcome Prediction Model

Prediction models were trained on the Discovery cohort using linear discriminant analysis (LDA)^21^ implemented in the caret package, version 6.0-80.^22^ LDA attempts to discriminate between classes by identifying linear boundaries around clusters of tumors using 5-hmC levels. Normalized batch- and sex-corrected read counts for each gene were treated as a feature in the model. Features were filtered for nonzero variance and correlation coefficient less than 40%. Models were trained with 10-fold cross-validation optimized for area under the curve. Sensitivity and specificity were determined for the validation cohort.^22^ Matthew’s correlation coefficients (MCC) were generated to determine the fit of the model.^23^ Similar to other correlation statistics, a score of 1 denotes a perfect model. Kaplan-Meier curves and hazard ratios were generated according to predicted event and survival status in Prism, version 6.0h (GraphPad Software, San Diego, CA).

## RESULTS

### Patient Characteristics and Outcome

The Discovery cohort included 51 patients with LR (n = 24), IR (n = 11), and HR (n = 16) tumors. The Validation cohort included 38 patients with LR (n = 13), IR (n = 12), and HR (n = 13) tumors. The COG cohort included 20 patients with HR neuroblastoma. Patient and tumor characteristics are summarized in Table 1 and detailed in the Data Supplement. Outcome was evaluable for all patients except one LR patient who transferred care after initial resection. For the 36 LR patients with evaluable outcomes, the 5-year event-free survival (EFS) was 94.4% (SE, 3.8%) and the overall survival (OS) was 100%. IR patients had an estimated 5-year EFS rate of 76.1% (SE, 9.5%) and OS rate of 95.7% (SE, 4.3%), and the HR patients had estimated 5-year EFS and OS rates of 44.8% (SE, 9.2%) and 52.5% (SE, 9.6%), respectively.

**TABLE 1.**
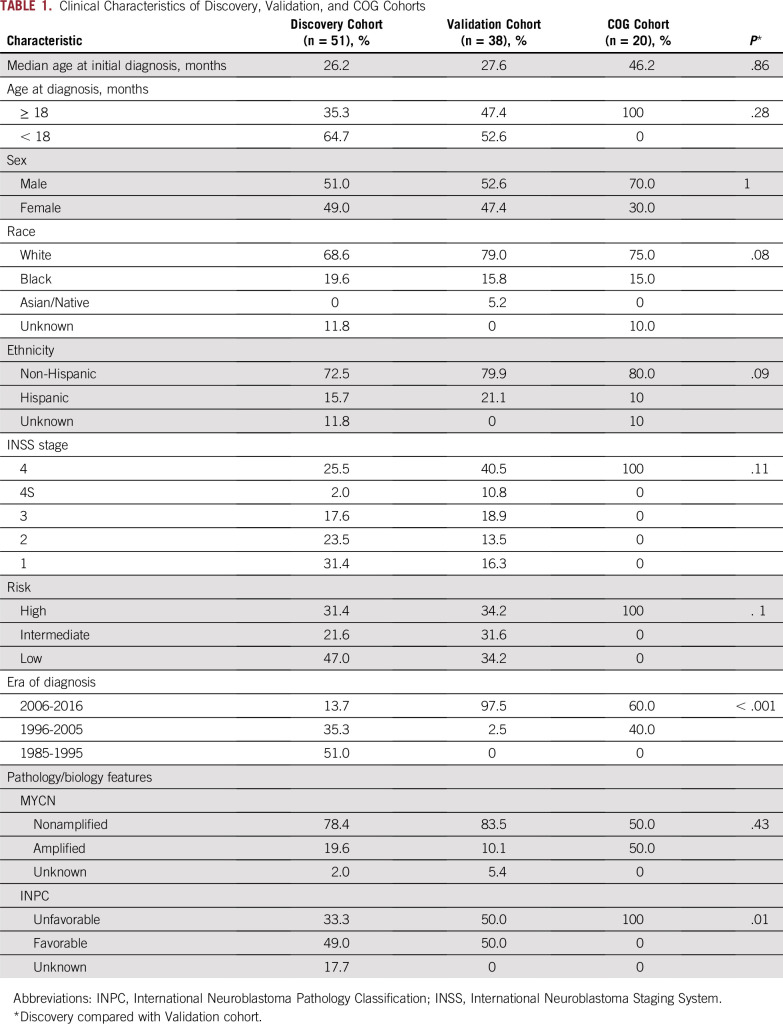
Clinical Characteristics of Discovery, Validation, and COG Cohorts

### Nano-hmC-Seal Profiling Distribution by Functional Genomic Features

**FIG 1.**
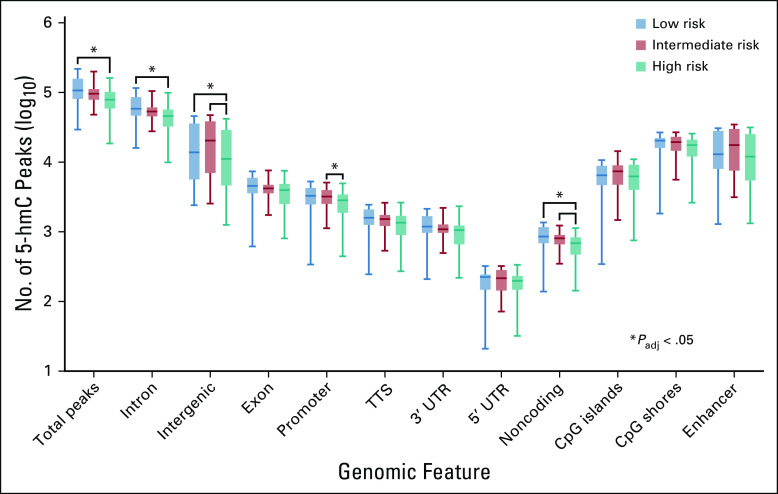
5-hmC by genomic feature in neuroblastoma tumors according to risk group. Overall, the low-risk (blue) and intermediate risk (red) tumors had more 5-hmC than high-risk tumors (green). 5-hmC was annotated with hypergeometric optimization of motif enrichment software. Comparisons between risk groups were assessed by the pairwise *t* tests with Benjamini-Hochburg correction. TTS, transcription termination site.

Total 5-hmC was higher in the 37 LR and 23 IR tumors compared with the 29 HR tumors (false discovery rate [FDR] = 0.03 and 0.08, respectively; Fig 1). 5-hmC was most common in intronic and intergenic regions, with statistically significant increased 5-hmC in LR compared with HR tumors in intronic (FDR = 0.04) and intergenic regions (FDR = 0.01), consistent with previous studies that have reported decreased 5-hmC in more aggressive malignancies.^24^ 5-hmC was commonly found in CpG islands, CpG shores, and enhancer regions, accounting for 4.2%, 11.6%, and 9.1% of all regions, respectively.

### 5-hmC Profiles Associated With Neuroblastoma Risk Group

**FIG 2.**
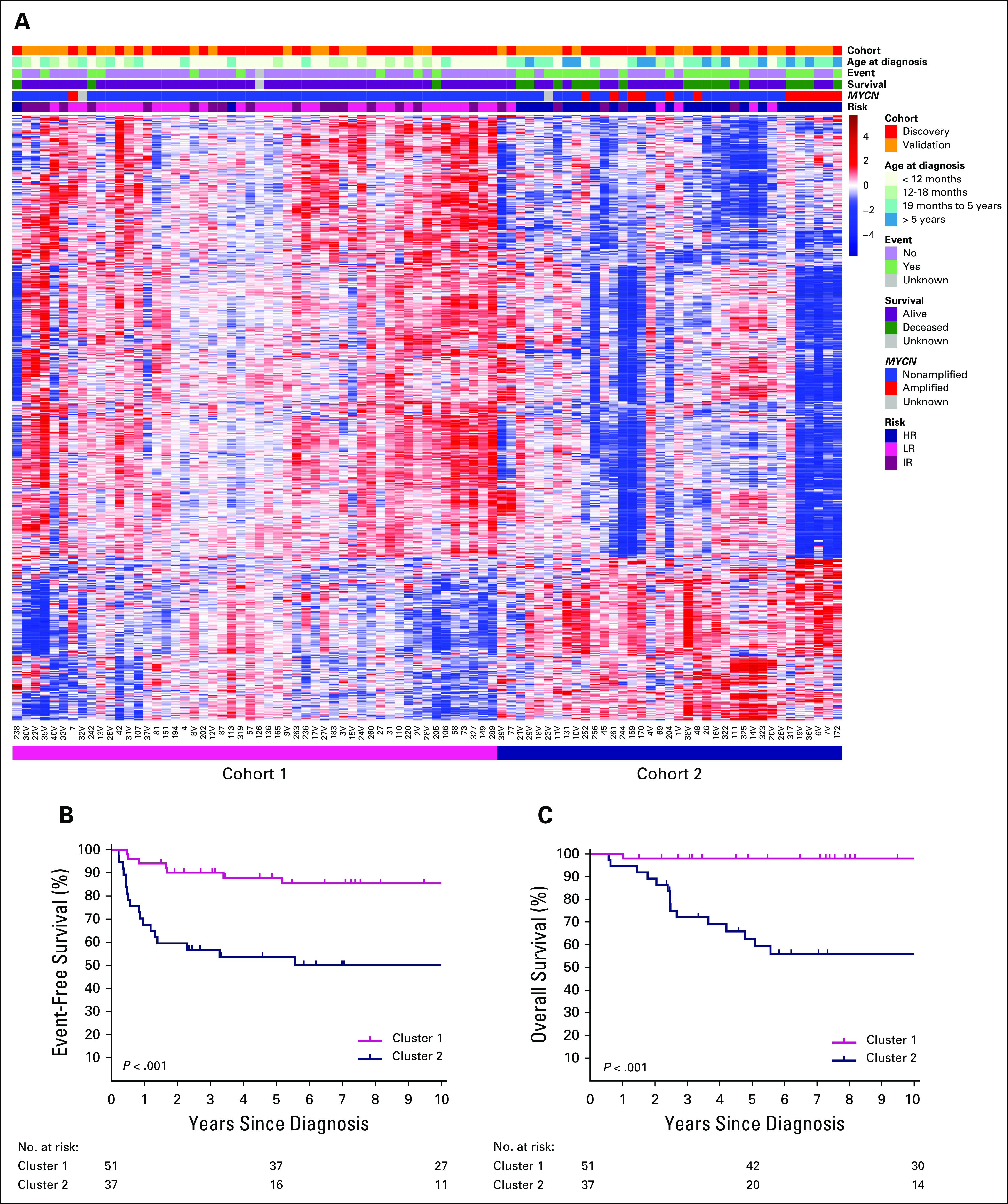
Clustering of Discovery and Validation cohorts using genes with differential 5-hmC identified in the Discovery cohort. (A) Two main clusters that were highly correlated with prognostic markers, clinical risk group, and outcome were identified. Cluster 1 included 86% of the tumors from LR patients, whereas 89% of the tumors from HR patients were in cluster 2. (B) The 5-year event-free survival was significantly inferior for patients in cluster 2 compared with cluster 1 (53.6% *v* 87.6%, respectively; *P* < .001). (C) The 5-year OS was also inferior for cluster 2 compared with cluster 1 patients (62% *v* 97.7%, respectively; *P* < .001). HR, high risk; IR, intermediate risk; LR, low risk.

Analysis of the 16 HR and 24 LR tumors from the Discovery cohort identified 577 unique genes with differential 5-hmC between risk groups (Data Supplement). Because of the unique biology conferred by amplification of the *MYCN* oncogene,^25^ we compared the accumulation of gene body 5-hmC in the eight *MYCN*-amplified HR tumors with accumulation in the 24 LR tumors, and then analyzed 5-hmC levels in the eight non-*MYCN*–amplified HR tumors compared with the same LR tumors (Data Supplement). Hierarchical clustering of the pooled Discovery and Validation tumors (n = 89 LR, IR, and HR tumors) using these 577 genes revealed two primary clusters that correlated highly with prognostic markers, clinical risk group, and outcome (Fig 2A). Cluster 1 included 86% of the tumors from LR patients, whereas 89% of the tumors from HR patients were in cluster 2. As expected, based on the high percentage of HR tumors in cluster 2, 5-year EFS was significantly worse for this subset of patients compared with cluster 1 (53.6% *v* 87.6%; *P* < .001; Fig 2B). Five-year OS was also inferior for cluster 2 patients (62% *v* 97.7%; *P* < .001; Fig 2C). Among the five IR patients in cluster 2, three had an event and two died, which highlights the potential for 5-hmC profiles to refine risk stratification. Of the 18 IR patients in cluster 1, 14 (78%) were event free at time of last follow-up, and three of four IR patients with an event were successfully treated and are alive.

### 5-hmC as a Marker of Chromosomal Copy Number

**FIG 3.**
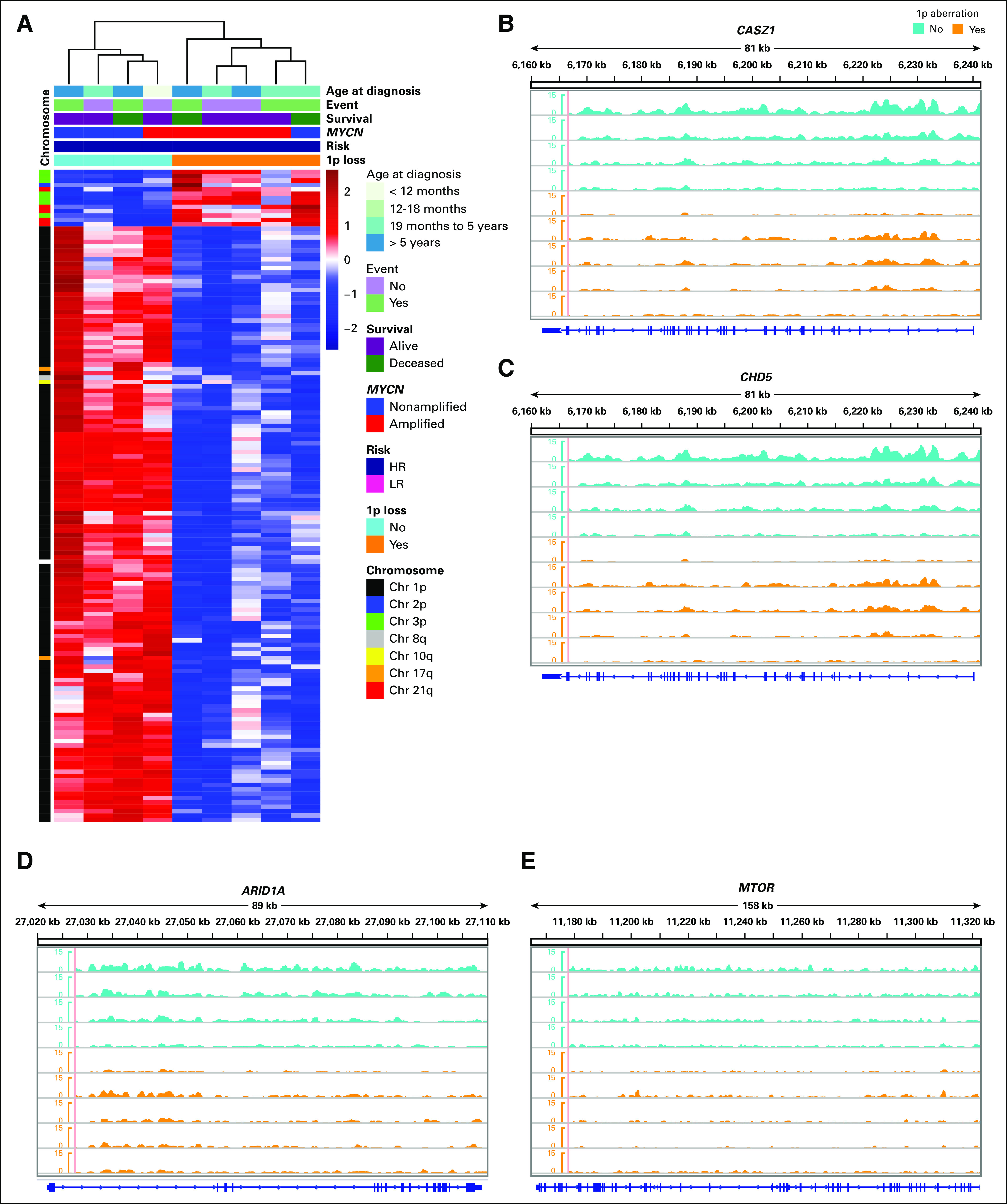
5-hmC differences in HR tumors according to chromosome 1p status. (A) Clustering of nine HR tumors with known copy number at chromosome 1p. Genes with differential 5-hmC predominate on chromosome 1p, highlighting the underlying chromosomal aberration. (B-E) Genome browser views of 5-hmC signals detected in four genes (*CHD5*, *CASZ1*, *ARID1A*, *MTOR*) with known biologic functions in neuroblastoma, illustrating decreased 5-hmC in tumors with chromosome 1p aberrations that likely modulate tumor biology. HR, high risk; IR, intermediate risk; LR, low risk.

Chromosomal aberrations are prognostic in neuroblastoma and are correlated with *MYCN* status.^26^ To determine the relationship between 5-hmC profiles and copy number, we performed differential 5-hmC analyses between 52 cluster 1 and 37 cluster 2 tumors (Data Supplement) and assessed the chromosomal location of the 3,320 identified genes. Random sampling showed the 3,320 genes were significantly enriched for mapping to chromosome 1p (*P* < .001). In the cluster 2 tumors, a decrease in 5-hmC was detected in 92.1% of the 240 genes that mapped to 1p. Because chromosomal aberrations vary according to *MYCN* status, we conducted copy number analysis on nine available HR tumors from the Discovery cohort, five of which were *MYCN* amplified and four were non-*MYCN* amplified. Chromosome 1p loss was detected in five tumors, four of which were *MYCN* amplified. Comparison of the tumors with versus without 1p loss identified 148 genes with differential levels of 5-hmC (FDR < 0.1; Fig 3A). Interestingly, 131 (88.5%) of the 148 genes were located on chromosome 1p, suggesting Nano-hmC-Seal has the potential to identify copy number alterations within tumor subsets. Chromosome 1p contains several genes with important biologic functions in neuroblastoma, including *CHD5*, *CASZ1*, *ARID1A*, and *MTOR* (Fig 3B-3E).^27^ Increased 5-hmC was also detected in several additional genes in tumors with chromosome 1p loss, including tumor-promoting genes *ABCG1* and *S100B* mapping to chromosome 21q.

### Gene Expression, 5-hmC Levels, and Cellular Pathway Analysis

**FIG 4.**
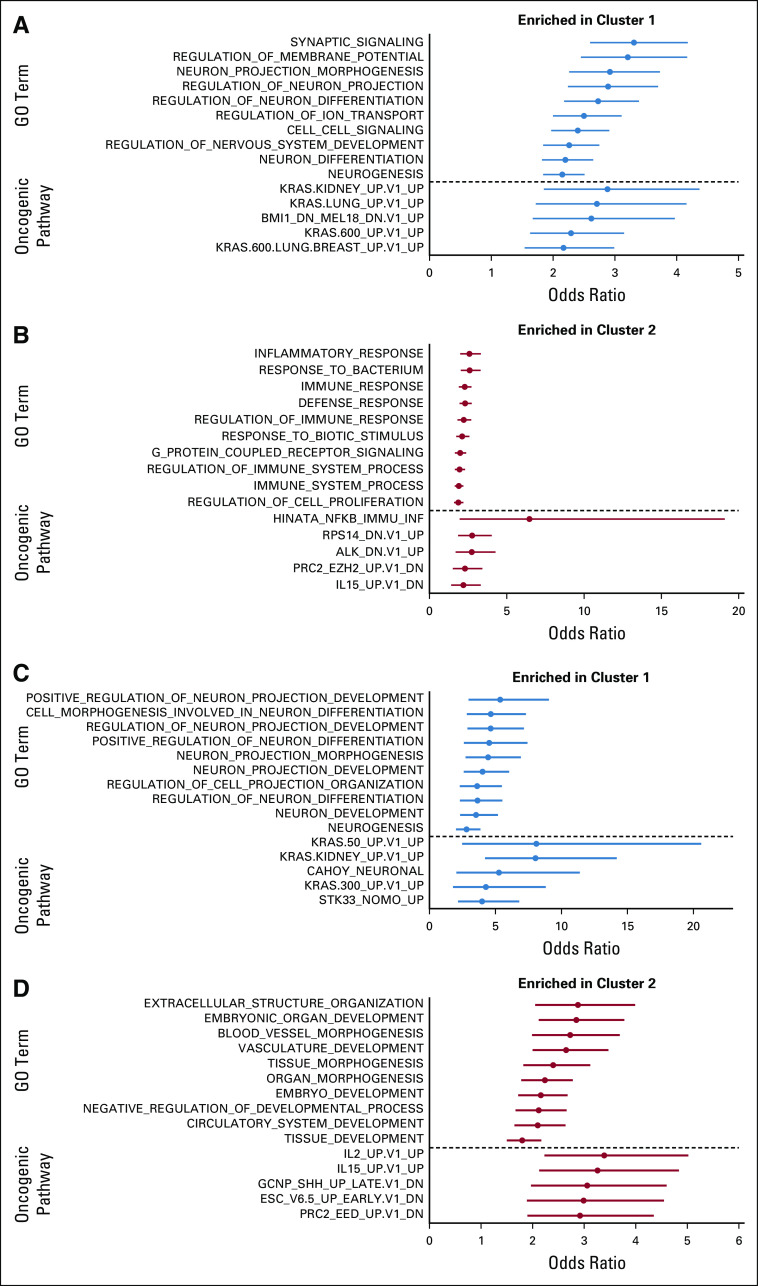
Pathway enrichment analysis for differentially regulated genes from cluster 1 and cluster 2 tumors. (A) Genes with increased 5-hmC in cluster 1 tumors were enriched for gene ontology (GO) pathways of neuronal differentiation and oncogenic signatures of activated KRAS signaling and genes that are regulated by BMI1 and MEL18. (B) Cluster 2 tumors had increased 5-hmC in genes enriched for GO pathways of an inflammatory response. These genes also showed increased 5-hmC in genes involved in activation of the PRC2 complex. (C) Genes with increased 5-hmC and expression in cluster 1 tumors were also enriched for neuronal differentiation and KRAS activation. (D) Cluster 2 tumors had increased 5-hmC and expression in genes enriched for pathways of embryo development, morphogenesis, IL-2, IL-15, and the PRC2 complex.

To further explore the biology of cluster 1 and cluster 2 tumors, we examined the 3,320 genes with differential 5-hmC between these tumors (Data Supplement). Genes with elevated 5-hmC in cluster 1, compared with cluster 2 tumors, showed enrichment for gene ontology (GO) pathways of neuronal differentiation, similar to prior analysis of genes expressed in LR tumors.^28^ Also identified were oncogenic signatures of activated KRAS signaling and suppression of BMI1 and MEL18, integral components of the polycomb repressive complex 1 (PRC1). In contrast, cluster 2 tumors had elevated 5-hmC compared with cluster 1 tumors in genes enriched for pathways of an inflammatory response and oncogenic signatures including activation of EZH2 and the PRC2 complex, transcriptional networks reported previously in HR tumors (Fig 4A and 4B).^29^

Because 5-hmC is a marker of transcriptional activation, we performed RNA-seq on one HR, 11 IR, and 17 LR tumors from cluster 1 and 17 HR and three LR tumors from cluster 2 (Data Supplement). Differentially expressed genes were identified between the two clusters. Genes with increased expression in cluster 1 were highly represented for neuronal differentiation. In cluster 2, overexpressed genes were enriched for pathways of embryo development and morphogenesis (Data Supplement).

Differential 5-hmC was assessed in these same 48 tumors, identifying 2,722 genes that met significance and filtering criteria (Data Supplement). To estimate the degree of sharing between genes with differential 5-hmC and expression, we extracted the RNA-seq differential fold change and calculated the *P* value for each of the genes with significantly differential 5-hmC.^30^ Using the π1 statistic,^24^ we estimated that 62% of the 2,722 genes with significantly differential 5-hmC were also differentially expressed between the two clusters. Then, using a stricter threshold of FDR < 0.05 and filtering by top 10% in fold change, we identified 283 genes that had significantly increased 5-hmC and expression in cluster 1 and 1,184 genes that had significantly increased 5-hmC and expression in cluster 2 (*P* for overlap < .001). Genes in cluster 1 tumors were again enriched for GO pathways of neuronal differentiation and oncogenic signatures of activated KRAS signaling. Cluster 2 tumors were enriched for GO pathways of embryo development and morphogenesis and oncogenic signatures including activation of IL-2, IL-15, and the PRC2 complex (Fig 4C and 4D).

### 5-hmC as a Predictive Biomarker of Outcome in HR Patients

**FIG 5.**
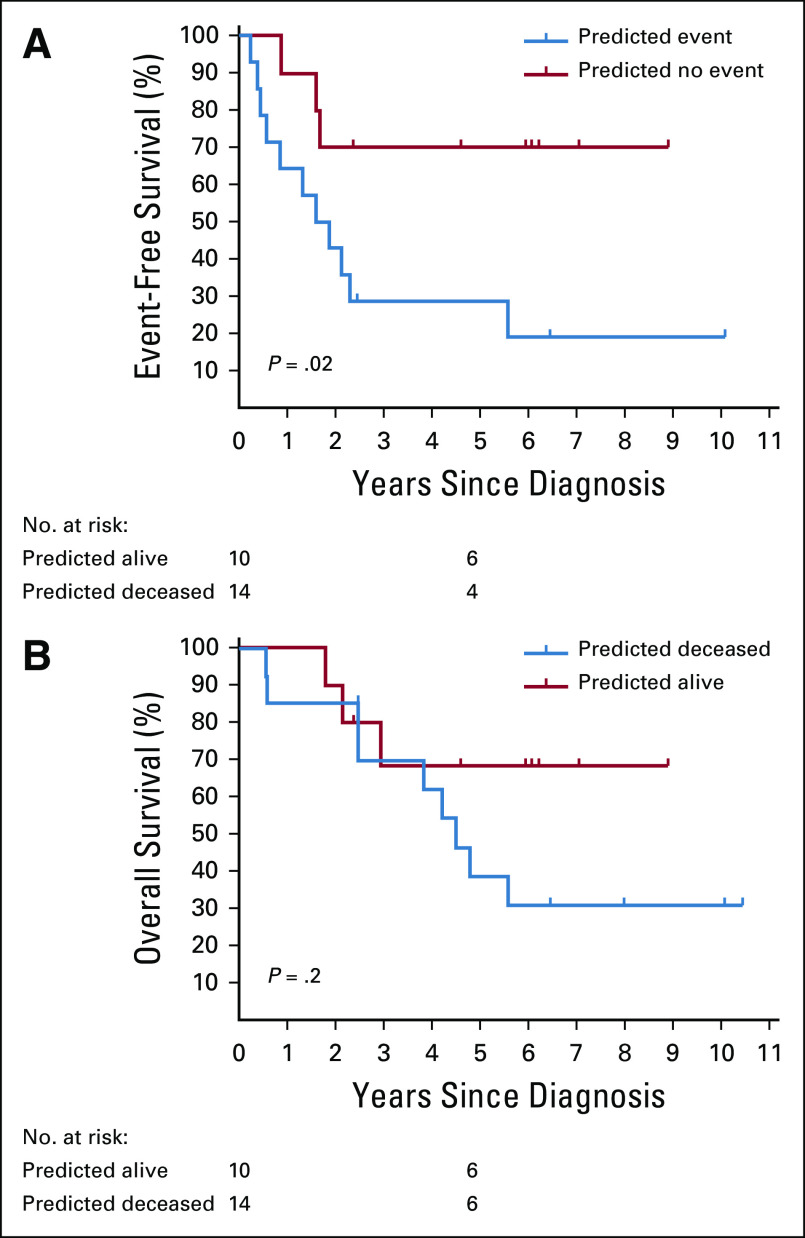
Kaplan-Meier curves of high-risk patients from the combined Validation and COG cohorts according to event-free or overall survival status. Event-free or overall survival status was predicted using a linear discriminant analysis model trained on high-risk patients from the Discovery cohort optimized for overall survival. (A) Event-free survival was significantly better for patients who were predicted not to have an event, although no significant difference in (B) overall survival was observed in those predicted to be alive or not.

We performed an exploratory evaluation of 5-hmC levels as a prognostic marker for patients classified as HR (Data Supplement). The LDA model trained on HR patients in the Discovery cohort based on their OS correctly predicted EFS in 18 of 24 patients from the combined Validation and COG cohorts. They were assigned to a favorable or unfavorable outcome group with a sensitivity of 70%, specificity of 79%, positive predictive value of 78.6% (95% CI, 57.8% to 90.8%), and MCC of 0.49. The 5-year EFS for patients predicted to be in the favorable outcome group was 70% (95% CI, 32.9% to 89.2%) versus 28.6% (95% CI, 8.8% to 52.3%) for those in the unfavorable outcome group with a hazard ratio of 3.8 (95% CI, 1.2 to 9.6; *P* = .02; Fig 5A). The model correctly predicted OS for 16 of 24 patients into a favorable or unfavorable outcome group with a sensitivity of 58%, specificity of 75%, positive predictive value of 64.3% (95% CI, 46.1% to 79.1%), and MCC of 0.34. The 5-year OS for patients predicted to be in the favorable group was 68.6% (95% CI, 30.5% to 88.7%) versus 39% (95% CI, 14.3% to 63.3%) for those in the unfavorable group with a hazard ratio of 2.3 (95% CI, 0.68 to 6.7; *P* = .2; Fig 5B).

## DISCUSSION

Using the novel Nano-hmC-Seal technology, we identified distinct 5-hmC profiles correlated with established prognostic factors, clinical risk groups, and outcome of patients with neuroblastoma. Hierarchical clustering based on differential 5-hmC levels in 577 genes separated the tumors into two main clusters. Cluster 1 contained 32 of the 37 LR tumors analyzed, 18 IR tumors, and 3 HR tumors. In contrast, cluster 2 contained the majority of tumors that were clinically classified as HR (27 of the 29 evaluated), five IR tumors, and five LR tumors. Consistent with the large number of HR tumors, 5-year EFS and OS were significantly inferior for cluster 2 patients compared with those in cluster 1. Three of the five IR patients in cluster 2 relapsed and two died, suggesting 5-hmC profiles may provide additional prognostic information in these non-high–risk cohorts.

We also demonstrate 5-hmC levels may be prognostic of EFS but not OS in HR patients. Patients with relapsed neuroblastoma frequently survive for extended periods before succumbing to disease, and EFS is often used as a primary end point in clinical trials for this reason.^31^ Higher levels of total 5-hmC, measured by immunohistochemistry or mass spectrometry, have been reported to be associated with less aggressive tumor behavior and improved survival in adult patients with cancer.^32-34^ In addition, cell-free DNA 5-hmC signatures are diagnostic biomarkers of leukemia, esophageal, pancreatic, liver, and colon cancers.^5,35^ To our knowledge, this is the first study to show that gene-specific 5-hmC profiles may also be prognostic of patient outcomes.

Chromosomal copy number variation is commonly detected in neuroblastoma, and patterns of chromosomal aberrations are strongly prognostic of survival.^26^ We show the potential for 5-hmC profiling to identify segmental chromosomal aberrations without the need for additional testing. Our results indicate that in tumors with 1p loss, decreased levels of 5-hmC were detected in the majority of genes that map to chromosome 1p. Consistent with these findings, others have reported increases of the repressive 5-methylcytosine modification on genes on chromosome 1p in neuroblastoma with identical copy number alterations.^36^ Although we identified large chromosomal aberrations, Nano-hmC-Seal is unlikely to replace direct copy-number analysis to identify small, segmental aberrations.^37^

There is increasing evidence that 5-hmC plays an important role in activating transcription, and that variation in 5-hmC positively correlates with gene expression levels.^4^ We found many genes with both increased expression and 5-hmC, which is consistent with other tumor types.^3,4^ In the favorable cluster 1 tumors, we found GO enrichment of neuronal differentiation from the genes with increased in 5-hmC and expression. Neuronal differentiation is a hallmark of LR tumors,^38^ and studies indicate HRAS expression drives this phenotype.^39^ Pathway analysis also suggested a role for the PRC1 complex, which promotes gene silencing by monoubiquitinating histone 2A.^40^ Additional efforts are needed to confirm our findings, which could provide additional evidence for targeting BMI1 in neuroblastoma.^41^

In the unfavorable cluster 2 tumors, we found that the genes with increased 5-hmC and expression were enriched for PRC2 complex target genes. Upregulation of EZH2, an integral part of the PRC2 complex, leads to the transcriptional repression of differentiation genes and maintains stem-like cell properties.^42^ Recent studies have established the PRC2 complex has high-binding affinity for methylated cytosines,^43^ making a direct connection between histone and DNA epigenetics.^44^ In addition, EZH2 is integral to the biology of HR neuroblastoma and considered a potential therapeutic target in the disease.^45^

Nano-hmC-Seal can rapidly identify 5-hmC levels genomewide, using a cost-effective sequencing approach that requires low sample input and no specialized biospecimen handling. Although a number of prognostic gene expression signatures have been validated in neuroblastoma,^28,46-48^ these biomarkers have not been integrated into the clinic, in part because of challenges obtaining high-quality RNA from diagnostic tumor samples. Moreover, neuroblastoma tumorigenesis and phenotype are thought to be driven by epigenetic reprogramming,^49^ driving the need for new epigenetic biomarkers. 5-hmC profiling could provide information on factors that determine the clinical behavior of neuroblastoma: *MYCN* status, copy-number alterations, and transcriptional networks, using one simple assay. Efforts are underway to test the prognostic value of 5-hmC profiles in a prospective clinical trial using both tumor tissue and cell-free DNA.
